# Comprehensive Analysis of Event‐Related Potentials of Response Inhibition: The Role of Negative Urgency and Compulsivity

**DOI:** 10.1111/psyp.70000

**Published:** 2025-02-04

**Authors:** Verena Wüllhorst, Raoul Wüllhorst, Rebecca Overmeyer, Tanja Endrass

**Affiliations:** ^1^ Faculty of Psychology, Institute of Clinical Psychology and Psychotherapy Technische Universtität Dresden Dresden Germany

**Keywords:** compulsivity, EEG, impulsivity, negative urgency, response inhibition, single‐trial‐regression analyses

## Abstract

Behavioral and neural correlates of response inhibition are assumed to relate to impulsivity and compulsivity, but findings are inconsistent, possibly due to prior research studying these dimensions in isolation. Negative urgency, the tendency to act impulsive under negative affect, and compulsivity relate to various mental disorders and are assumed to reflect deficits in inhibitory control. However, few studies have examined how response inhibition relates to negative urgency, compulsivity, or their interaction. To address this gap, we conducted a comprehensive analysis of the behavioral and neural correlates of response inhibition and their associations with negative urgency and compulsivity. We examined 233 participants who performed a stop‐signal task while electroencephalography was recorded. The analysis involved single‐trial regression and latency analyses to explore the relationships with self‐reported negative urgency and compulsivity. Stop‐signal reaction times (SSRTs) and negative urgency were associated with an attenuated P3 effect contrasting successful stop versus go trials. Crucially, longer SSRT was associated with reduced P1 amplitudes (on successful and failed stops) and a later onset and peak of the P3. Interestingly, the opposite pattern was observed for higher negative urgency with higher P1 amplitudes and an earlier P3 onset and peak in successful stop trials. Associations with compulsivity were not observed. Considering early sensorimotor processes and latency effects are important to capture differences between negative urgency and SSRT. Higher stop‐signal‐related P1 amplitudes and a faster action cancellation process may compensate reduced P3‐related activity in high negative urgency.

## Impact statement

With event‐related potentials (ERPs) in a large sample, we add a new evidence on the complex association between neural processes of inhibitory control and negative urgency, and thus go one‐step further than studying isolated ERP components. We show enhanced early stop‐signal‐related P1 amplitudes and faster recruitment of the P3 that compensate for reduced P3 amplitudes in individuals reporting high negative urgency.

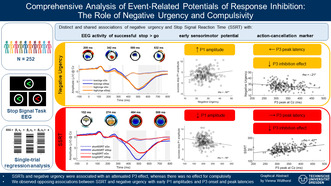

## 1 Introduction

Cognitive control flexibly supports goal‐directed behavior, for instance, by initiating and inhibiting actions. In turn, impaired response inhibition may result in negative urgency, that is, rash and uncontrolled actions or responses when experiencing negative affect, or compulsivity, that is, persistent behavior with problems in terminating actions (Chikara, Su, and Ko 2019; Dalley et al. 2011; Whiteside and Lynam 2001). The objective of the current study was to investigate the association of response inhibition and its neural correlates, using electroencephalography (EEG), with the traits of negative urgency and compulsivity.

### 1.1 Neural Correlates of Action‐Stopping

Response inhibition comprises both suppressing motor responses before initiating an action, assessed with go/no‐go tasks, and canceling already initiated actions, assessed with stop signal tasks (SSTs, e.g., Verbruggen and Logan 2008). As the process of action‐stopping is fast, the high temporal resolution of EEG is well‐suited for examining the neural implementation of response inhibition. Particularly, the N2/P3 complex has been related to response inhibition (Huster et al. 2020; Kok et al. 2004). The P3, a positive deflection between 300 and 500 ms after the stop signal (De Jong et al. 1990; Enriquez‐Geppert et al. 2010; Kok et al. 2004; Wessel and Aron 2015), is associated with activity in the inhibitory control network (inferior frontal gyrus, pre‐supplementary motor area, and anterior insula, Baumeister et al. 2014; Kenemans 2015). Importantly, action‐stopping is not only reflected by the amplitude of ERPs but also their latency, which indicates the speed of neural processing. As such, the onset of the P3 has been suggested as an indicator of action‐stopping (Wessel and Aron 2015), and a meta‐analysis has shown that P3 onset and peak latencies are more closely related with inhibitory performance than its amplitude (Huster et al. 2020). In contrast to robust P3 findings across task versions, earlier ERPs differ between visual and auditory SSTs (Carrillo‐de‐la‐Peña, Bonilla, and González‐Villar 2019). In auditory stop tasks, stop signals elicit a stop N1 (Kenemans 2015; Skippen et al. 2020), which peaks around 100 ms after the stop signal and is reliably larger on successful than failed stop trials. In contrast, visual stop tasks generate the N2, a negative fronto‐central deflection approximately 200 ms after the stop signal (Kenemans 2015; Kok et al. 2004; Raud et al. 2020), but its properties suggest different interpretations. Some studies found the N2 to be larger on infrequent stop compared to go trials (aggregated stopping‐effect, e.g., Enriquez‐Geppert et al. 2010), indicating a possible conflict effect. Others interpret the N2 as a signal triggering the inhibition process (e.g., Senderecka 2016). When comparing failed versus successful stop trials, some observed no difference in amplitudes between these conditions (Dhir et al. 2022), some found larger N2 amplitudes on successful versus failed trials (e.g., Schmajuk et al. 2006), and others reported a larger N2 on failed versus successful trials (Carrillo‐de‐la‐Peña, Bonilla, and González‐Villar 2019; Greenhouse and Wessel 2013; Kok et al. 2004; Senderecka 2016).

Recent studies using visual SSTs showed early stop‐signal‐related components: a centroparietal P1 that was larger for successful versus failed trials (Rueda‐Delgado et al. 2021), and a larger N1 at similar electrode sites (Kenemans et al. 2023). It was suggested that theses attentional and sensorimotor processes, reflected by early ERPs, might facilitate stopping, for example, through faster initiation of the cancellation process (Hampshire and Sharp 2015; Kenemans 2015; Logan and Cowan 1984), or by enabling adaptive behavior via an active performance monitoring system (Norman et al. 2019). Consequently, the investigation of early ERPs may provide insights into how fluctuations in early processes influence action‐stopping.

### 1.2 Alterations of Action‐Stopping in Psychopathology

Deficits in response inhibition, measured with the stop‐signal reaction time (SSRT), have been observed in substance use disorders (Smith et al. 2014) as well as obsessive‐compulsive disorder (OCD, Mar et al. 2022). Although impulsivity, compulsivity, and prolonged SSRT have each been linked to maladaptive behaviors in daily life (e.g., Jones et al. 2018; Lee, Hoppenbrouwers, and Franken 2019), consistent associations between these factors are lacking (e.g., Sharma, Markon, and Clark 2014). The absence of associations between SSRT and impulsivity (e.g., Dhir et al. 2022; Shen, Lee, and Chen 2014) may result from the use of composite scores for impulsivity, which can obscure the effects of specific sub‐facets of impulsivity. The UPPS Impulsive Behavior Scale (UPPS; Whiteside and Lynam 2001) reflects the multidimensional structure of impulsive behaviors, differentiating urgency (i.e., to act rashly under negative emotions), lack of perseverance (i.e., the inability to complete tasks), lack of premeditation (i.e., the tendency to act without thinking and planning), and sensation seeking (i.e., the tendency to seek out new and exciting experiences). Among these, only (negative) urgency is specifically defined in terms of problems with inhibitory control (Whiteside and Lynam 2001). Consistent with this, only negative urgency has been linked to failures in action‐stopping both in general contexts (Basar et al. 2010; Whiteside and Lynam 2001; Wilbertz et al. 2014) and threatening contexts (Roxburgh, White, and Cornwell 2022), emphasizing the relevance of examining negative urgency specifically in relation to action‐stopping. Regarding compulsivity, some studies have found no association between compulsivity and SSRT (Dhir et al. 2022; Lei et al. 2015; Linkovski et al. 2013), while Kloft, Riesel, and Kathmann (2019) showed that high scores of compulsivity were related to long SSRT in OCD.

ERPs of SST performance offer precise temporal information about the neural processes involved in response inhibition. For instance, the P3 component of the stop ERP has been linked to inhibitory control, that is, reduced P3 was shown to be associated with longer SSRTs (Huster et al. 2020; Kenemans et al. 2023) or larger N1/P1 amplitudes have been associated with successful stop responses (Bekker et al. 2005; Rueda‐Delgado et al. 2021; Skippen et al. 2020). Therefore, ERPs allow to disentangle the timing of the following effects: inhibitory performance (SSRT), and self‐reported negative urgency as well as compulsivity, assessed with the Obsessive‐Compulsive Inventory‐Revised (OCI‐R; Foa et al. 2002). However, only few studies investigated the relationship between inhibition‐related ERPs and negative urgency as well as compulsivity and results are inconsistent. Dück et al. (2023) found no significant effects of impulsivity, negative urgency, compulsivity, or their interaction on ERPs of response inhibition in a go/no‐go task. Similarly, Dhir et al. (2022) observed no association between these traits and the inhibition‐related P3 in successful stop compared to go trials, but the interaction of compulsivity and impulsivity accounted for variance in failed stop N2 and P3. Other studies reported that impulsivity was associated with reduced P3 amplitudes (Shen, Lee, and Chen 2014) and diminished inhibitory activity during fMRI (Wilbertz et al. 2014). In contrast, some researchers reported enhanced P3 amplitudes for higher impulsivity (Dimoska and Johnstone 2007; Lansbergen et al. 2007). Associations of impulsivity with inhibitory deficits and a reduced nogo‐N2 in individuals with internet gaming disorder have been proposed to reflect the high cognitive demands involved in inhibiting actions (Kim et al. 2017). Thus, it is challenging to draw conclusions about the association between neural correlates of action‐stopping and impulsivity or compulsivity. Discrepancies in findings may result from variations in sample composition, small sample sizes, and the neglect of specific facets of impulsivity, such as negative urgency. Furthermore, interpretating previous results is complicated by the potential disappearance of between‐person effects when accounting for varying stop signal delays (SSDs), that is, trial‐wise fluctuations in task difficulty (D'Alberto et al. 2018). Finally, the P1 (~110–180 ms, posterior) predicted problematic alcohol use over and above N2 and P3 activity in a visual SST (O'Halloran et al. 2019), emphasizing the importance of investigating early ERPs in relation to impulsivity and compulsivity, too.

The current study had two main objectives: First, we aimed to investigate the ERP correlates associated with different aspects of action‐stopping, including successful inhibition, and the differences between successful and failed inhibition. This included examining stop‐signal‐related P1 amplitudes and P3 onset, peak latency, and amplitude to investigate early processes and inhibition‐related ERPs. Second, we sought to examine how impulsivity, compulsivity, and their interaction relate to neural correlates of successful inhibition, focusing on the N2/P3 complex and P1 component. We expected that negative urgency (rather than an impulsivity composite score) and compulsivity would be associated with prolonged SSRT, indicating impaired inhibitory performance. We hypothesized that SSRT, negative urgency, and compulsivity would be associated with a reduced successful inhibition‐P3 effect (successful stop vs. go trials) and delayed P3 onset and peak latencies. We explored whether the interaction of negative urgency and compulsivity modulates the effects of successful versus failed inhibition, and examined the associations of early stopping effects (related to P1) with negative urgency, compulsivity, their interaction, and SSRT.

## 2 Methods

### 2.1 Participants and Procedure

We recruited 252 participants from the general population such that a wide range of impulsivity and compulsivity scores are represented in our sample. In an online survey, impulsivity scores were assessed with the Barratt Impulsiveness Scale (Patton, Stanford, and Barratt 1995), and compulsivity scores with the OCI‐R (Foa et al. 2002; Gönner, Leonhart, and Ecker 2007). Afterward, participants were selected based on questionnaire scores and screened in a telephone interview for inclusion and exclusion criteria of the study. All participants had normal or corrected‐to‐normal vision, did not report any neurological disorders or head trauma, and were German native speakers. The exclusion criteria were the use of psychotropic substances within the past 3 months, a history of bipolar disorder, borderline personality disorder, psychotic episodes, severe alcohol use disorder (> 5 DSM‐5 criteria), use of illicit substances of more than twice a year per substance, and lifetime use of cannabis of more than twice a month. Further, individuals with current eating disorders or severe episodes of major depression were excluded. Participants were excluded after testing because they (*N* = 3) used psychotropic substances 24 h prior to testing or they (*N* = 16) did not have two valid blocks in the SST [i.e., *p*(respond|signal) between 0.4 and 0.6; see section ‘Stop Signal Task’ for details]. The resulting sample included *n* = 233 participants with a mean age of 24.8 years (SD = 4.5; 50% female), and then, 95% of the participants had completed advanced education degrees.

The study consisted of two laboratory sessions and a week of ecological momentary assessment. During the second session, participants performed a visual SST and completed questionnaires on impulsivity and compulsivity. Other tasks and questionnaires were obtained but are not part of this report. All participants gave informed consent and received the 80–100 Euro for participation. The study was conducted in accordance with the ethical guidelines of the Declaration of Helsinki, and the ethics committee of the Technische Universität approved the study procedures (EK 372092017).

### 2.2 Measurements

#### 2.2.1 Negative Urgency

We used the 45‐item UPPS (Schmidt et al. 2008; Whiteside and Lynam 2001) to assess negative urgency, lack of premeditation, lack of perseverance, and sensation seeking. Higher scores on the Likert‐type scale (1 = strongly disagree to 4 = strongly agree) indicate high expressions on the respective facet. All subscales exhibit good internal consistencies ranging from Cronbach's *α* = 0.80 to 0.85 (Schmidt et al. 2008).

#### 2.2.2 Compulsivity

The OCI‐R (Foa et al. 2002; Gönner, Leonhart, and Ecker 2007) assesses obsessive‐compulsive symptom severity. We used the sum score of all 18 items to operationalize compulsivity, which shows good reliability in German (Cronbach's *α* = 0.85, Gönner, Leonhart, and Ecker 2007). Descriptive results of the self‐report measures, as well as associations among the different subscales of the UPPS and OCI‐R are depicted in Table S1.

### 2.3 Stop Signal Task

A visual SST was used and consisted of 25 practice trials and up to four blocks, each consisting of 128 trials. During the task, a white circle presented on a gray background was always visible. Each block included 96 (75%) go trials, on which the circle remained empty for 300–500 ms and then a white arrow pointing to the right or left appeared in the center of the circle for a duration of 1000 ms. Participants were asked to respond as quickly and accurately as possible with their index fingers according to the direction of the arrow. The arrows were 2.5‐cm wide and high, viewing distance approximately 73 cm, resulting in a visual angle of 1.6°. Each block included 32 (25%) stop trials, on which—after a delay relative to the go onset (SSD)—the arrow turned red (the stop signal), indicating that the response should be stopped. The SSD adapted in a trial‐wise fashion, to ensure that participants approached 50% *p*(respond|signal) by increasing or decreasing SSD by 50 ms after successful or failed inhibitions, respectively. There were four different starting SSDs (100, 150, 200, and 250 ms), each active on 25% of stop trials and tracked by its own adaptation algorithm (Aron and Poldrack 2006). The minimum and maximum SSDs were set to 50 and 350 ms, respectively. Go and stop trials were presented in pseudorandom order with the following constraints: The first three trials were go trials, left and right arrows occurred equally often within go and stop trials, stop trials and arrow direction did not repeat more than twice, and the maximum number of go trials between two stop trials was five. The intertrial interval relative to go/stop‐stimulus offset lasted 400–600 ms. After each block, participants received feedback to respond faster if *p*(respond|signal) < 0.4, more accurately if *p*(respond|signal) > 0.6, or unchanged if *p*(respond|signal) ≥ 0.4 and ≤ 0.6. The task stopped after the participants completed two blocks with a stopping probability rate between 0.4 and 0.6 or after a maximum number of four blocks. Thus, all participants included in the analysis have two valid blocks with a total of 256 trials (64 stop trials). Completing the task took between 10 and 20 min.

### 2.4 Electroencephalogram Recording

EEG data were recorded with 61 AG/AgCl electrodes using two 32‐channel BrainAmp amplifiers (Brain Products GmbH, Munich, Germany) and an EasyCap electrode cap with equidistant electrode locations (EasyCap GmbH, Herrsching‐Breitbrunn, Germany). The impedances were kept below 10 kΩ. The ground and reference electrodes were mounted next to Fz (at AFF1h and AFF2h, theta/phi spherical coordinates: −58/78 and 58/78). At the lower back, one electrode recorded the electrocardiogram, and below the left and right eye, two electrodes were placed to capture eye movements in combination with electrodes mounted in the cap. Data were continuously registered at a sampling frequency of 500 Hz.

### 2.5 Data Analyses

#### 2.5.1 Behavioral and Correlational Analyses

The integration method with replacement of go omissions was used to estimate SSRT (Logan and Cowan 1984; Verbruggen et al. 2019). Briefly, the point at which the stop process finishes (*n*th RT) was assumed to be approximately the point at which the integral of the RT distribution of all go trials equals the probability to respond to the stop signal [*p*(respond|signal)]. Go omissions were replaced by the maximum RT of successful go trials. The SSRT estimate resulted from subtracting the mean SSD from the nth percentile of the go RT distribution. Trials with an RT below 80 ms were excluded from all analyses. Further, analyses were only performed for participants with two valid blocks with *p*(respond|signal) between 0.4 and 0.6 (*n* = 235) and shorter go than failed stop RT (*n* = 233).

In correlational analyses, data points deviating more than 3 SDs from the mean were regarded as outliers and removed from the respective analysis (*n* = 3, SSRT). We corrected with the Bonferroni method for multiple testing and with the Bonferroni–Holm method for analyzing associations between compulsivity and sub‐facets of impulsivity.

#### 2.5.2 EEG Analyses

Data were analyzed using EEGLAB 14.1 (Delorme and Makeig 2004). The EEG was re‐referenced to the common average reference and band‐pass filtered with 0.1–30 Hz. Ocular artifacts were removed using an adaptive mixture independent component analysis, as implemented in EEGLAB. Go‐ and stop‐related epochs spanning from 500 ms before to 1000 ms after stimulus onset (go, stop) were obtained separately. Epochs that contained deviations greater than 5 SDs of the mean probability distribution were automatically rejected, as were epochs with early responses (< 80 ms) or (> 600 ms). Independent components reflecting artifacts were rejected manually by the first author. Subsequently, we applied baseline correction using the individual mean value of the interval between −500 and 0 ms prior the go onset or −200 and 0 ms prior the stop onset.

##### 2.5.2.1 Onset and Peak Latency Analyses

Following Wessel and Aron (2015), we determined the onset of the P3 as the time at which ERPs significantly differed between stop and go trials with sample‐wise truth‐label switching Monte Carlo permutation tests (10,000 iterations). Since SSDs changed throughout the task, and shorter versus longer SSDs on previous stop trials may influence motor preparation, we determined ERPs for go trials that were matched to stop trials based on SSD expectancy. As such, we randomly selected one out of all go trials that occurred after a specific stop trial, and before the next stop trial, to match that specific previous stop trial and its associated SSD. Go‐ERPs were then epoched to the onset of this fictive SSD, that is, at the time point when a potential stop stimulus was expected to have occurred. For more details regarding trial matching, refer to Wessel and Aron (2015). We determined P3 onsets separately for successful and failed stop trials. We chose Cz based on the literature (Huster et al. 2020; Wessel and Aron 2015) and Pz as an electrode of interest, as we observed centroparietally distributed effects in the successful and successful versus failed inhibition model, reported below under First‐level single‐trial regression results. We quantified the peak latency by defining the P3 peak as the most positive deflection between 200 and 600 ms after the stop signal.

##### 2.5.2.2 Associations Within the Process of Action‐Stopping

**FIGURE 1:**
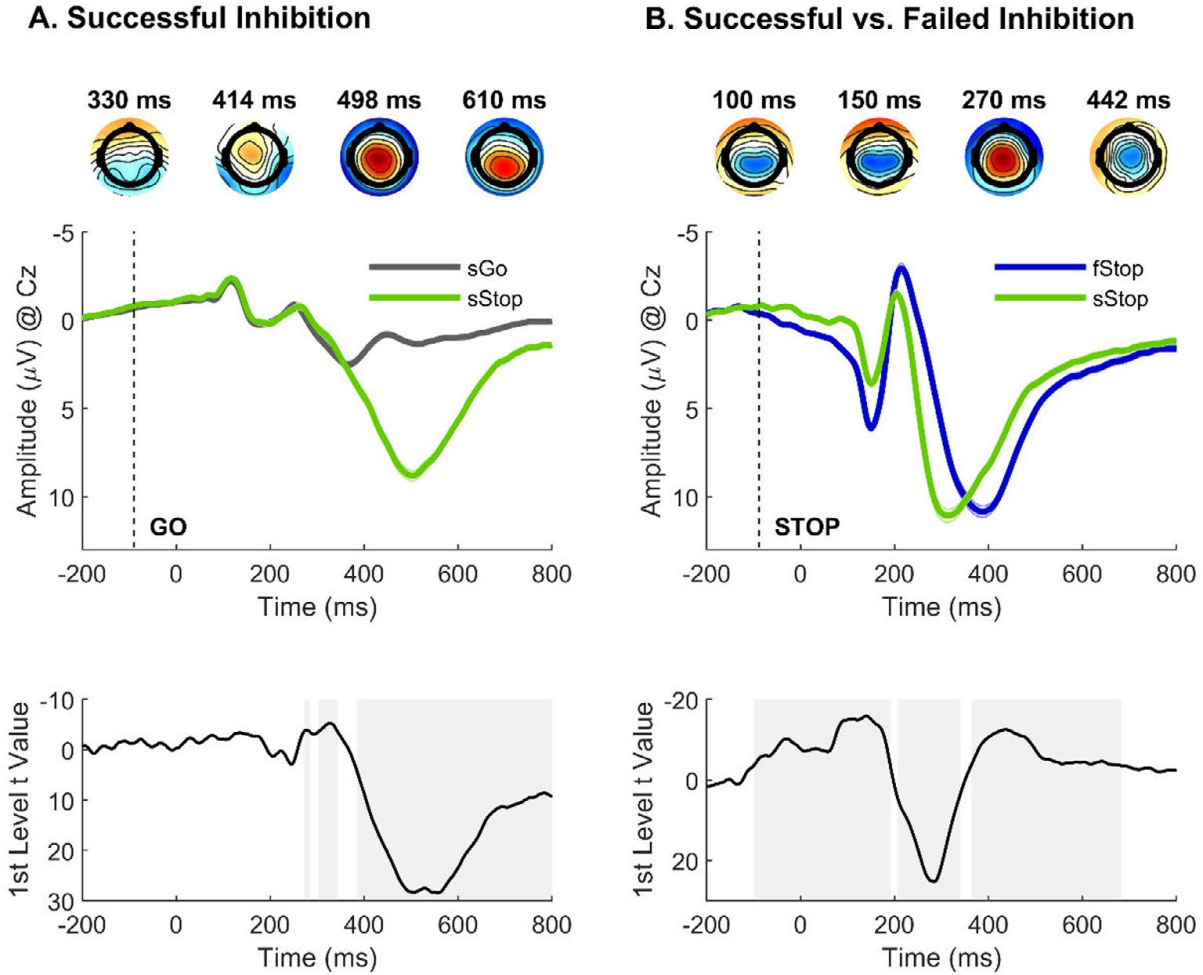
First‐level main regression effects of successful inhibition and successful versus failed inhibition. (A) Successful inhibition effect of model (1): successful stop versus go trials at Cz. (B) Successful versus failed inhibition effect of model (2): successful versus failed stop trials at Cz. At the top, topographical maps represent significant associations between EEG activity and the regressor in question [*t* values, red: positive, blue: negative, masked at critical *p* = 5 × 10^−4^ for model (1) and *p* = 4 × 10^−4^ for model (2)]. Time courses of (A) go‐locked and (B) stop‐locked EEG activity are presented in the center. At the bottom, trajectories of the *t* values for the regressor trial type are shown (gray shades reflect significance at the respective critical *p*). Note that successful stop trials were coded as 1, whereas go trials in model (1) and failed stop trials in model (2) were coded as −1. Therefore, positive *t* values reflect more positive, and negative *t* values reflect less positive, amplitudes on successful stops. fStop, failed stop trials; sGo, successful go trials; sStop, successful stop trials.

We aimed to investigate the associations between EEG activity during the inhibition process, particularly successful inhibition. We tested whether successful and failed stop‐signal‐related P1 amplitudes were correlated with P3 onset, peak latencies, and amplitudes. We extracted P1 mean amplitudes between 130 and 170 ms at CP1 (the stop‐signal‐related P1 effect in the successful vs. failed inhibition model was maximal at 150 ms, see Figure 1B) separately for successful and failed stop trials. P3 amplitude was quantified as the mean amplitude between 200 and 600 ms at Cz.

##### 2.5.2.3 Single‐Trial Regression Analyses

We used MATLAB version 2022a (MathWorks) to conduct robust single‐trial regression analyses (Fischer et al. 2016; Fischer and Ullsperger 2013). On the first level, we computed two GLMs predicting EEG activity at all electrodes (*n* = 63) and time points, yielding individual beta (and corresponding t) values per regressor. We then tested each of these *t* values against zero with two‐tailed one‐sample *t* tests to determine statistical significance, while correcting for multiple testing with the false discovery rate (FDR; Benjamini and Yekutieli 2001) procedure (fdr_*q* = 0.001). We obtained the corresponding trajectories of the *t* values and topographies of the effects, critical *p* values, and an overall *R* ^2^. The models described below were the (1) successful inhibition model and (2) successful versus failed inhibition model.

All GLMs were based on following the formula:

(1) $$\mathrm{EEG}=\beta_{0}+\beta_{1}\times \text{trial type}+\beta_{2}\times \text{response side}+\text{Error}$$

with trial type reflecting the predictor of main interest (specific to each model as described below) and response side (left, right) as regressor of no interest.

1. *Successful inhibition model*: Trial type was defined as successful stop versus go (coded as 1 and −1).
2. *Successful* versus *failed inhibition model*: Trial type was defined as successful stop Versus failed stop (coded as 1 and −1).

Whereas model (2) used EEG activity locked to the onset of the stop signal, model (1) used EEG activity locked to the onset of the go signal. To establish a procedure similar to the latency analysis (Wessel and Aron 2015), we added SSD and the interaction of SSD and trial type as regressors of no interest to model (1). As the go trials have no respective SSD, we used the last SSD of the preceding stop trial as a proxy. We deviated from this procedure for model (2) by excluding SSD as a regressor, because SSD was highly associated with successful stopping, resulting in problems of multicollinearity. In order to make it easier to map results to their respective analysis, we used “effect” whenever we refer to results from the regression analysis, and “amplitudes” or “latencies” in reference to latency, control, or post hoc analysis, in which we quantified ERPs.

We chose the successful inhibition model (1) to analyze inhibition effects, which is in line with a wide range of EEG (Dieterich et al. 2021; Huster et al. 2017; Wessel and Aron 2015), EMG (e.g., Raud and Huster 2017), and MRI (Hildebrandt et al. 2023; Hu et al. 2016) studies using the SST. We used the successful versus failed inhibition model (2) to analyze early processes on stop trials that differed between successful and failed stops. We included both models because the contrast of successful versus failed stop trials potentially underestimates the role of saliency processes on motor inhibition (Hampshire and Sharp 2015; Hu et al. 2016). Further, a large body of research revealed shorter latencies of the P3 onset and peak on successful compared to failed stop trials (e.g., Kok et al. 2004; Wessel and Aron 2015). Therefore, single‐trial regression analyses (which rely on contrasts time point by time point) on the successful versus failed stop contrast are only informative for EEG activity prior to latency divergence.

##### 2.5.2.4 Second‐Level Analyses

We examined the dimensional between‐subjects effects based on the following GLM:

(2) $$\text{First level}\;t=\beta_{0}+\beta_{1}\times \mathrm{neg}.\text{urgency}+\beta_{2}\times \text{compulsivity}+\beta_{3}\times \mathrm{neg}.\;\text{urgency}\times \text{compulsivity}+\beta_{4}\times \text{SSRT}+\text{Error}$$

On the second level, the *t* values of the first‐level effects served as dependent variables in robust multiple regression models. For this purpose, we used the contrast successful stop versus go from model (1) and the contrast successful versus failed stop from model (2). Negative urgency, compulsivity, the interaction negative urgency × compulsivity, and SSRT served as predictors (Equation 2), yielding beta coefficients and corresponding *t* values, the latter of which we report in this manuscript. Nonsignificant predictors were iteratively removed in a backward stepwise fashion. We normalized questionnaire data (range: 0–1) and demeaned scores for the interaction of negative urgency and compulsivity. We set the threshold to correct for multiple testing by dividing *α* = 0.05 by the number of predictors per model.

#### 2.5.3 Control Analyses

As the time interval between go and stop stimuli is not only short, but also differs because of the varying SSD on stop trials, the final stop‐ERPs and inhibition‐related effects might be distorted by overlapping go‐ and movement‐related activity (e.g., Woldorff 1993). To address this problem, we conducted control analyses with the MATLAB toolbox *unfold* (Ehinger and Dimigen 2019) to disentangle stop signals from go‐ and motor‐related EEG activity by a linear deconvolution algorithm. We excluded failed go trials in accordance with the successful inhibition model (1) and the successful versus failed inhibition model (2). We specified a generalized linear model with three predictors: go stimulus, go‐related button press (left vs. right side), and stopping (response vs. no response) with the following formula to predict EEG activity with a time expansion (for a detailed description, see Ehinger and Dimigen 2019):

$$\mathrm{EEG}\sim 1\left(\mathrm{go}\;\text{stimulus}\right)+\mathrm{cat}\left(\mathrm{go}\;\text{response}\right)+\mathrm{cat}\left(\text{stopping}\right)$$

with go stimuli entered as an intercept term, and cat() indicating that the predictors go response (left vs. right side) and stopping (response/failed vs. no response/success) were dummy‐coded. The unfold analysis revealed the model coefficients (betas) of each predictor as waveforms (i.e., regression‐ERPs [rERPs]), capturing only the partial effect of the respective predictor. For instance, the partial failed stop‐rERP betas correspond to the difference wave of failed stop‐ERP minus go‐ERP and response‐ERP‐related activity. To test whether the results of the single‐trial regression analysis (see above) remained after controlling for these confounds, we proceeded as follows. To recreate our single‐trial regression results, we performed robust regression analyses on each time point of adjusted EEG activity at the electrode yielding maximal effects for the trial‐type regressor in the first‐level analyses:

$${\mathrm{EEG}}_{\mathrm{adj}}=\beta_{0}+\beta_{1}\times \text{trial type}+\text{Error}$$

On the second level, we used the difference scores [partial successful stop‐rERP betas minus partial go‐rERP betas to emulate model (1), and partial successful stop‐rERP betas minus partial failed stop‐rERP betas to emulate model (2)] to compute robust regression analyses at each time point with negative urgency, compulsivity, or SSRT as predictors. Further, we extracted mean amplitudes and latencies of the adjusted EEG activity in exactly those intervals where the stop‐signal‐related P1, N2, and P3 occurred. We conducted correlational analyses and *t* tests with the rERPs to examine whether we can replicate the remaining results (e.g., different modulations of P3 peak latency by negative urgency, or larger P1 amplitude in failed compared to successful stops).

#### 2.5.4 Exploratory Analysis

Based on the recent findings of a visual stop N1 (Kenemans et al. 2023), we conducted an additional exploratory analysis to test for a similar effect in our data. We applied an analysis analogue to Kenemans et al. (2023) on the unfold‐corrected data at Cz, 90–120 ms where the visual stop N1 was maximal, with a poststimulus baseline correction of 0–50 ms, and tested for an amplitude difference between successful versus failed stop trials.

## 3 Results

### 3.1 Task Performance and Self‐Report

Task performance is reported in Table 1. SSD was not associated with SSRT (ρ = 0.08, *p* = 0.22). None of the inhibition‐related performance measures (SSRT, SSD, go omissions) were associated with negative urgency, compulsivity, or their interaction (see Supporting Information for correlation coefficients). These results did not change after excluding three participants with SSRTs in the outlier range. Within the questionnaire data, negative urgency was associated with compulsivity (ρ = 0.196, *p* < 0.01).

**TABLE 1:** Descriptive results of task behavior and measures of inhibition.

	Mean (SD)	Min	Max
Measures of inhibition			
SSRT (ms)	202.74 (26.92)	133.37	307.03
SSD (ms)	178.76 (22.51)	125.78	228.13
*p* (successful response | stop signal)	0.51 (0.05)	0.41	0.59
Go trials			
Go RT (ms)	394.77 (34.38)	328.33	510.15
Go error rate	0.02 (0.02)	0	0.22
Go omission rate	0.002 (0.01)	0	0.05

Abbreviations: RT, reaction time; SSD, individual stop signal delay at the end of the task; SSRT, stop‐signal reaction time.

### 3.2 EEG Results

#### 3.2.1 Onset and Latency Effects

Results of the latency analyses showed that the onset and peak of the P3 at Cz occurred earlier on successful than failed stop trials, P3 onset, *t*(192) = −15.35, *p* < 0.001 (mean [SD] in successful stop trials = 268.21 [40.29], and in failed stop trials = 311.39 [45.24]), and P3 peak, *t*(231) = −13.56, *p* < 0.001 (mean [SD] in successful stop trials = 334.90 [47.77], and in failed stop trials = 384.40 [51.60]).

#### 3.2.2 First‐Level Single‐Trial Regression Effects

##### 3.2.2.1 Successful Inhibition Model

As shown in Figure 1A, our analysis revealed a significant main effect of successful inhibition (successful stop vs. go), characterized by a central to centroparietal distribution (from 384 ms onward, peak at Cz and 498 ms), *t*(230) = 28.20, *p* < 0.001. The regression weights associated with this effect were positive, indicating that the P3 was more positive on successful stop compared to go trials. The maximum *R* ^2^ = 0.172 was at 500 ms.

##### 3.2.2.2 Successful Versus Failed Inhibition Model

We observed a negative‐going EEG effect from 28 to 190 ms at the parietal electrodes (peak at CP1), corresponding to a reduced P1 on successful versus failed stop trials (see Figure 1B). To compare current effects with model (1), Figure 1B shows the effects at Cz with maximal *t* values at 100 ms, *t*(230) = −10.20, *p* < 0.001, and at 150 ms, *t*(230) = −10.62, *p* < 0.001. Note that the P1 peaked at 150 ms. Further, we observed a positive‐going EEG effect at the frontal electrodes (maximum at FCz), reflecting the N2 with more negative amplitudes on failed compared to successful stop trials. Note that the subsequent positive (peak at Cz and 270 ms, *t*[230] = 22.24, *p* < 0.001) and negative‐going (peak at Cz and 442 ms, *t*[230] = −12.08, *p* < 0.001) EEG effects are a result of latency differences (see Section 3.2.1) and should not be interpreted as amplitude effects.

#### 3.2.3 Association Between Early Stimulus Processing With Response Inhibition

The stop‐signal‐related P1 amplitude was associated with shorter P3 latency at Cz on successful stop trials, P3 onset (ρ = −0.17, *p* < 0.05), and P3 peak (ρ = −0.26, *p* < 0.001), as well as with shorter P3 onset latency on failed stop trials (ρ = −0.23, *p* < 0.01). Further, the P1 amplitude on successful stop trials was associated with larger P3 amplitudes on successful stop trials (ρ = 0.40, *p* < 0.001), as well as with larger P3 amplitudes on failed stop trials (ρ = 0.26, *p* < 0.001).

#### 3.2.4 Second‐Level Effects

##### 3.2.4.1 Modulations of P3 Onset and Peak Latencies

**FIGURE 2:**
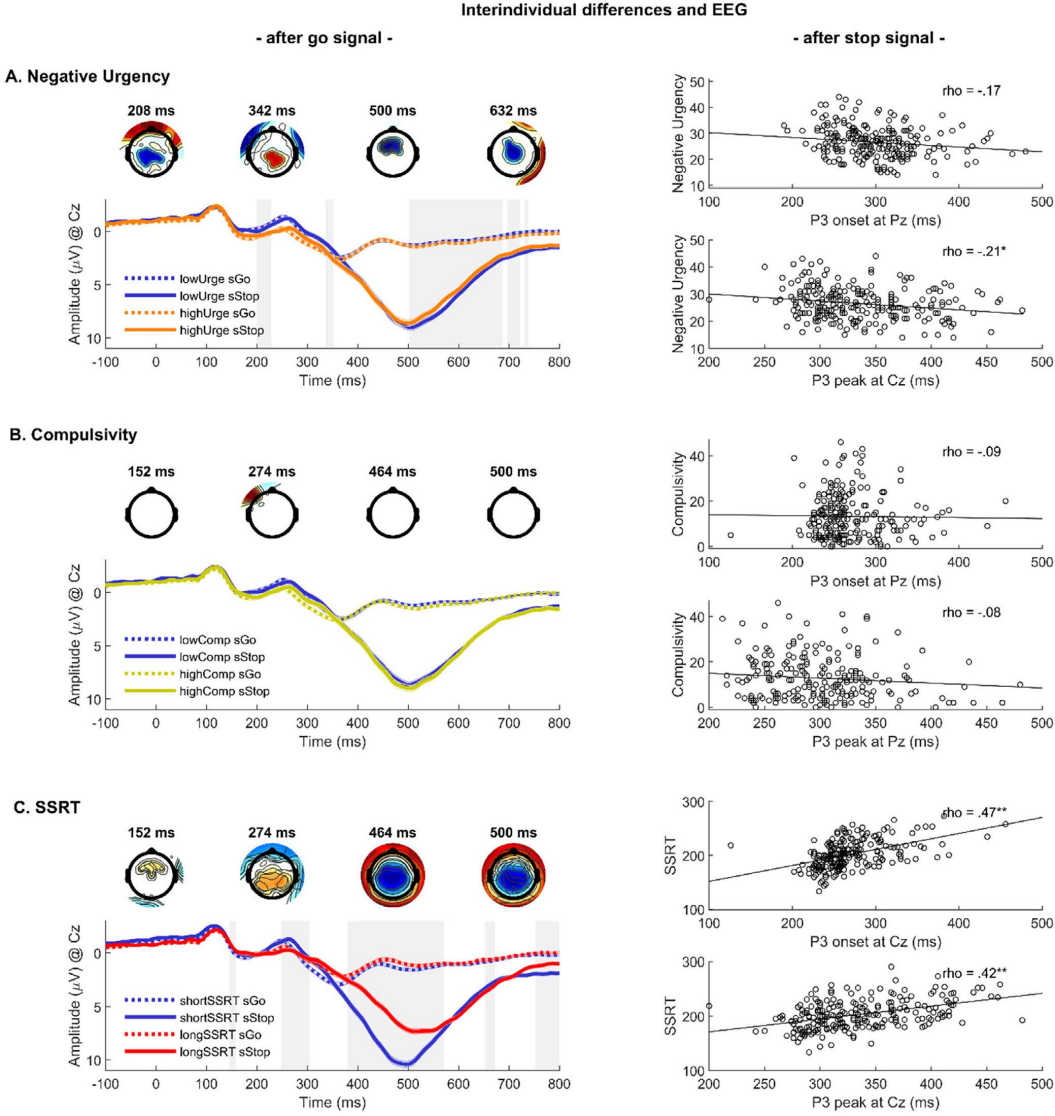
Second‐level effects of negative urgency, compulsivity, and SSRT on the successful inhibition contrast (successful stop vs. go). Second‐level effects of (A) negative urgency, (B) compulsivity (no significant association emerged), and (C) SSRT on the successful inhibition effect at Cz. On the left and top of each panel, topographical maps represent significant associations between first‐level effects and the regressor in question (*t* values, red: positive, blue: negative, masked at a corrected *p* = 0.0167). The waveforms of successful stop and go trials (comprising the successful inhibition effect) are shown below, with time point zero indicating the presentation of the go stimulus. For visualization purposes, waveforms are plotted for low versus high negative urgency (A), for low versus high compulsivity (B), and short versus long SSRTs (C) using the median split. Gray shades reflect significance at the respective critical *p*. On the right, scatterplots show associations of the regressors with P3 onset and peak latencies, respectively, selected for electrodes where correlations were the largest. Zero was set to the SSD, indicating the presentation of the stop stimulus. Critical *p* was 0.05/12 = 0.004 for correlational analyses. *p < 0.004, **p < 0.001. sGo, successful go trials; sStop, successful stop trials.

Negative urgency was associated with an earlier P3 peak at Cz (ρ = −0.21, *p* < 0.01), and at Pz (ρ = −0.20, *p* < 0.01), as well as P3 onset at Cz (ρ = −0.14, *p* < 0.05), and at Pz (ρ = −0.17, *p* < 0.05). Compulsivity was not associated with P3 onset or peak latency. Longer SSRTs were associated with later P3 onset, at Cz (ρ = 0.47, *p* < 0.001), Pz (ρ = 0.43, *p* < 0.001), as well as with longer latency of the P3 peak at Cz (ρ = 0.42, *p* < 0.001), and Pz (ρ = 0.34, *p* < 0.01). Scatterplots in Figure 2 show the results for electrodes with the strongest association of each predictor with P3 onset and peak.

##### 3.2.4.2 Modulations of Successful Inhibition Effects (Successful Stop vs. Go)

We found that higher negative urgency had a negative effect between 204 and 210 ms at Cz, maximum at 208 ms, *t*(230) = −2.46, *p* < 0.0167, and a positive effect between 334 and 352 ms at Cz, maximum at 342 ms, *t*(230) = 2.81, *p* < 0.0167. Importantly, the later P3 effect (difference between successful stop and go) was less pronounced with higher negative urgency, between 522 and 756 ms at Cz, maximum at 632 ms, *t*(230) = −2.97, *p* < 0.0167 (see Figure 2A).

Longer SSRT was associated with a larger effect (sStop vs. sGo) between 146 and 158 ms, peak at Cz and 152 ms, *t*(230) = 2.59, *p* < 0.0167, and 250–302 ms, peak at Cz at 274 ms *t*(230) = 3.76, *p* < 0.0167, reflecting a larger modulation of the P1 and N2. Further, SSRT had a negative effect from 382 to 566 ms at Cz, peak at 464 ms, *t*(230) = −9.53, *p* < 0.0167, reflecting a reduced P3 difference with longer SSRTs. Figure 2A,C shows the opposing association of negative urgency and SSRT with P3 onset and peak latencies, but similar associations with reduced P3 effects. Neither compulsivity (Figure 2B) nor the interaction of negative urgency and compulsivity showed significant effects.

##### 3.2.4.3 Modulation of Successful Versus Failed Inhibition Effects (Successful vs. Failed Stops)

**FIGURE 3:**
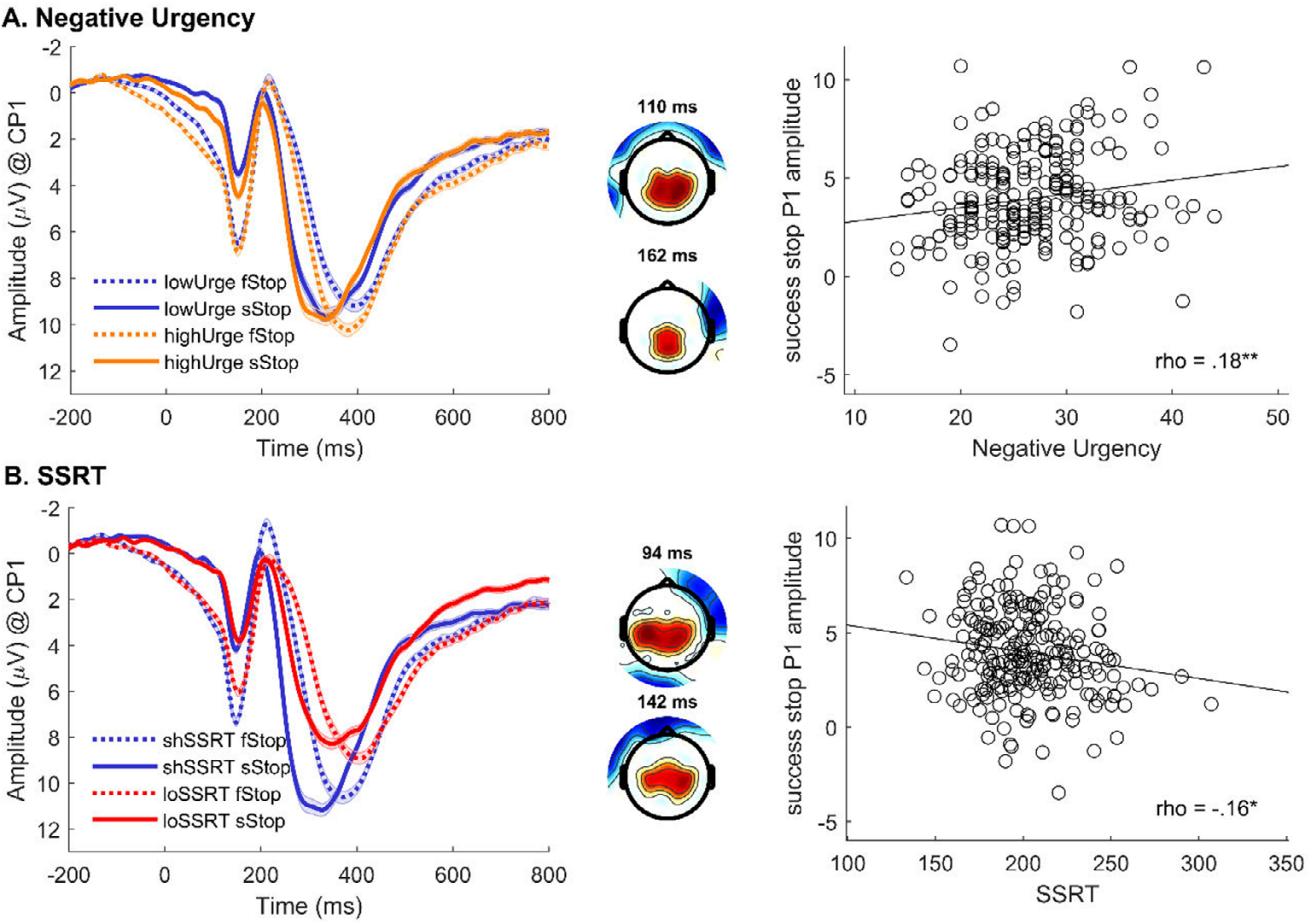
Associations of negative urgency and SSRT with the stop P1 effect. Modulation of the stop P1 effect by (A) negative urgency and (B) SSRT at CP1. On the left, waveforms of successful and failed stop trials are shown (for visualization purposes, waveforms are plotted for low vs. high negative urgency and short vs. long SSRTs after median split). In the middle, topographical maps represent significant associations between EEG activity and the regressor in question (*t* values, red: positive, blue: negative, masked at a corrected *p* = 0.025). Note that the positive second‐level *t* values displayed in these maps result from coding the P1 effect as a successful > failed (coded as 1 vs. −1) on the first level, which yielded negative first‐level *t* values in the P1 interval (cf. Figure 1B). Counterintuitively, positive second‐level *t* values in the current figure therefore indicate a smaller difference between successful and failed stops with increasing negative urgency and SSRT. On the right, scatterplots show the stop‐signal‐related P1 amplitudes for successful stop responses as a function of negative urgency and SSRT, respectively.

Negative urgency and SSRT were both associated with the P1 effect. Individuals with higher negative urgency showed a smaller difference between successful versus failed stops (first‐level P1 effect was less negative and therefore reduced) at CP1 between 98 and 124 ms, peaking at 110 ms, *t*(231) = 3.47, *p* < 0.025, and between 158 and 184 ms, peaking at 162 ms, *t*(231) = 2.53, *p* < 0.025, as shown in Figure 3A. Individuals with longer SSRT (i.e., reduced inhibitory performance) also showed a smaller difference between successful versus failed stops (first‐level P1 effect was less negative and therefore reduced) between 76 and 120 ms, peaking at 94 ms *t*(231) = 3.88, *p* < 0.025, and between 130 and 176 ms, peaking at 142 ms, *t*(231) = 3.38, *p* < 0.025 (see Figure 3B). Follow‐up analyses within trial types revealed that these apparent similarities were driven by opposite relationships: Higher negative urgency was associated with increased P1 amplitudes on successful (ρ = 0.18, *p* < 0.01), but not on failed stop trials (ρ = 0.03, *p* = 0.659). In contrast, longer SSRT was associated with decreased P1 amplitudes on both successful (ρ = −0.16, *p* = 0.013), and failed stop trials (ρ = −0.30, *p* < 0.01).

### 3.3 Control Analyses for Stop‐Signal‐Related ERPs

**FIGURE 4:**
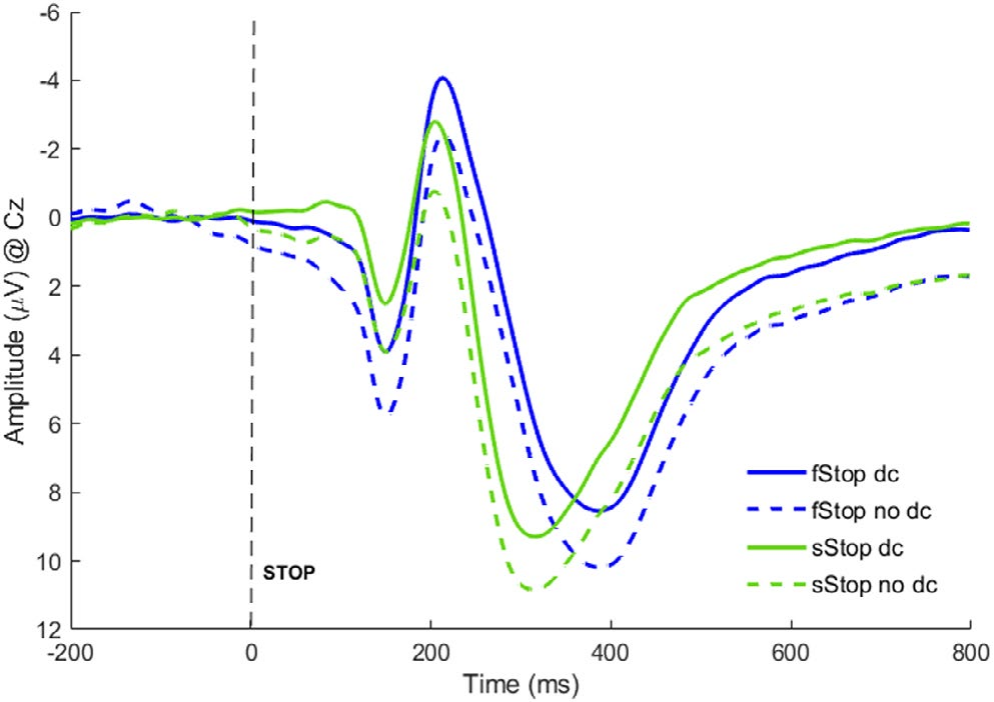
Comparison of EEG activity with and without deconvolution of go‐and motor‐ERP overlap on successful and failed stop trials. Time course of EEG activity for successful stop (sStop) and failed stop (fStop) trials with (dc) and without (no dc) applying the deconvolution procedure.

Removing go and movement overlap on stop trials significantly changed the size of all ERP amplitudes of interest, but not their latencies (see Figure 4), and for an overview of the mean amplitudes and statistics (see Table 2). Although deconvolution reduced the P1 and P3 amplitudes, the N2 amplitudes were larger. Deconvolution did not change the amplitude difference between successful and failed stop trials. As such, after applying the deconvolution analysis, the P1 amplitudes remained smaller on successful compared to failed stop trials at Cz, *t*(232) = 11.56, *p* < 0.001, and CP1, *t*(232) = 16.05, *p* < 0.001. The same applies to a larger N2 on failed compared to successful stop trials at Cz, *t*(232) = −7.52, *p* < 0.001. The P3 was larger for successful compared to failed stop trials (*t*(232) = 3.13, *p* < 0.01). In addition, the exploratory analysis of a potential visual stop N1 (maximal at Cz, 90–120 ms) with baseline correction of 0–50 ms revealed that amplitudes for successful stop trials were significantly more negative than failed stop trials (*t*(232) = 6.85, *p* < 0.001).

**TABLE 2:** Comparison of ERP mean amplitudes and peak latencies with and without the deconvolution procedure used.

ERP	Without dc		With dc		*t* value	*p*
	Mean	SD	Mean	SD		
Mean amplitudes						
P1—successful stop	3.95	2.34	1.65	1.56	22.04	< 0.001
P1—failed stop	6.17	2.60	3.44	1.98	23.90	< 0.001
N2—successful stop	−0.16	3.42	−2.36	2.73	15.93	< 0.001
N2—failed stop	−1.85	4.05	−3.70	3.82	13.32	< 0.001
P3—successful stop	8.79	3.61	6.93	3.11	14.26	< 0.001
P3—failed stop	8.82	3.94	7.12	3.34	14.07	< 0.001
Peak latencies						
P1—successful stop	154.49	9.51	154.52	9.71	0.21	0.830
P1—failed stop	147.10	14.46	147.27	13.93	0.29	0.770

Abbreviations: dc, deconvolution of data; SD, standard deviation.

### 3.4 Relationship of Negative Urgency and SSRT With the Adjusted ERPs

The control analyses using the adjusted ERPs are in line with the single‐trial regression results. Higher negative urgency significantly predicted a reduced difference between successful stop and go trials during the P3 interval at Cz between 402 and 460 ms, maximum at 422 ms, *β* = −0.28, *t*(231) = −2.86, *p* < 0.01, and 486–516 ms, maximum at 504 ms, *β* = −0.31, *t*(231) = −2.60, *p* ≤ 0.01. Further, higher negative urgency was significantly associated with a larger difference for successful stop versus go activity between 104 and 140 ms, maximum at 134 ms, *β* = 0.35, *t*(231) = 2.59, *p* ≤ 0.01.

Negative urgency was positively associated with the adjusted‐P1 on successful (ρ = 0.17, *p* < 0.01), but not on failed stop trials (ρ = 0.02, *p* = 0.721).

SSRT negatively predicted the difference between successful stop versus go trials at Cz between 16 and 72 ms, maximum at 32 ms, *β* = −2.80, *t*(231) = −2.95, *p* < 0.01, and between 130 and 142 ms, maximum at 136 ms, *β* = −1.61, *t*(231) = −2.73, *p* < 0.01, corresponding to the reduced P1 effect. This effect was followed by a positive association for the N2 between 168 and 200 ms, maximum at 180 ms, *β* = 1.87, *t*(231) = 4.06, *p* < 0.001, and a negative association for the P3 between 220 and 428 ms, maximum at 282 ms, *β* = −2.33, *t*(231) = −10.82, *p* < 0.001, and between 652 ms until the end of the analyzed time window (here 800 ms), maximum at 768 ms, *β* = −2.21, *t*(231) = −3.26, *p* < 0.01. SSRT was negatively associated with the adjusted‐P1 on successful (ρ = −0.16, *p* < 0.05) and failed stop trials (ρ = −0.28, *p* < 0.001).

## 4 Discussion

This study investigated neural correlates of response inhibition, and their associations with impulsivity, measured using SSRT and negative urgency, and compulsivity. Aside from validating findings on the N2/P3 complex widely reported in the response inhibition literature (e.g., Huster et al. 2020; Kok et al. 2004), we uncovered associations of the P1, potentially relevant for early sensorimotor and attentional activity, with markers of successful inhibition. Negative urgency predicted a reduced differentiation between successful stop and go trials for the P3, as well as *higher* P1 amplitudes for successful stop trials, followed by *earlier* successful stop P3 onset and peak. There was no evidence of an influence of compulsivity or an interaction of negative urgency and compulsivity on these processes. Similar to negative urgency, SSRT was related with the P3, showing reduced differentiation between successful stop and go trials as SSRT increased. However, higher SSRT predicted *lower* P1 amplitudes for successful and failed stop trials and *later* successful stop P3 onset and peak. In addition, early stop‐signal‐related P1 effects were related to P3 effects. These effects remained after removing the potential overlap of go and motor ERPs.

### 4.1 Neural Correlates of Action Cancellation and Associated Early Processing

The findings expanded our understanding of early sensorimotor and attentional activity interacting with markers of response inhibition. Successful inhibition may increase the signal‐to‐noise ratio in task‐relevant networks, resulting in the observed successful stop‐signal‐related P1 effect and potentially reflecting more effective processing (Klimesch 2011). As larger successful stop P1 amplitudes were associated with an earlier P3 onset and peak latency, and larger P3 amplitude, this connection may represent a feedforward sensory process that facilitates fast action cancellation (Slagter et al. 2016). However, further validation is required for this effect since it appears to differ from the existing literature on the role of attention in modulating early gating processes (e.g., Kenemans 2015). Contrary to expectations that higher activity on successful stop trials would enhance inhibitory control, we observed increased P1 amplitudes on failed compared to successful stop trials and on more difficult (long SSD) compared to easier trials (see Supporting Information: Section B). This speaks against the notion of an overall attention effect on the P1, which, according to the early selection hypothesis, should manifest in larger ERP amplitudes for attended stimuli (Di Russo, Martínez, and Hillyard 2003; Hillyard et al. 1973). Unlike Hillyard's findings, the SST induces two competing demands: (1) rapid response to a go stimulus, and (2) cancellation of this response upon a stop signal. Failures to stop may stem from prioritizing fast go responses over response stopping, from diminished attention, or both. The potential influence of diminished attention is supported by research showing a larger N1 for successful compared to failed stop trials in response to an auditory stop stimulus (Kenemans 2015; Kenemans et al. 2023; Skippen et al. 2020). Applying a later baseline (0 to 50 ms after the stop‐signal) reveals more negative amplitudes for successful compared to failed stop trials, consistent with an N1 effect in a visual SST paradigm (Kenemans et al. 2023). This visual stop N1 effect was larger for shorter SSRTs and would support the attention hypothesis. Thus, our interpretations remain speculative at this point but highlight the possibility of various early underlying processes with two conceivable interpretations. First, differences in amplitudes potentially indicate different strategic choices made by participants. Considering the role of the P1 as a perceptual gating marker (Herrmann and Knight 2001), known to be larger for complex targets (Klimesch 2011), the elevated P1 amplitude on failed stop trials may indicate larger costs and ineffective suppression of information. Second, the larger visual‐stop‐N1 effect could reflect increased early attentional processes on successful stop trials. Irrespective of a classification as P1 or visual‐stop‐N1 effect, these novel findings seem to be compatible with the Pause process of the recently postulated Pause‐Then‐Cancel model of human action‐stopping (Diesburg and Wessel 2021). Besides canceling the initiated action, the model postulates a complementary Pause process, reflecting a global inhibitory mechanism that suppresses the motor output of the go response via a hyper‐direct cortico‐basal ganglia pathway (reactive inhibition). Interestingly, key markers of this Pause process, such as motor suppression (Raud and Huster 2017), coincide with the stop‐signal‐related P1 or visual‐stop N1 effect (~150 ms after the stop signal). This supports our assumption that early ERPs may serve as additional signatures for the neural implementation of the Pause process. However, this assumption needs further validation, and the role of attention on early ERPs needs clarification.

### 4.2 Modulating the Process of Action Cancellation

As expected, the reduced successful inhibition P3 effect was associated with longer SSRT and higher negative urgency. The finding on negative urgency differs from results from Lansbergen et al. (2007) who found a larger P3 in individuals with high self‐reported impulsivity. However, it aligns with other findings in externalizing psychopathology, high trait impulsivity, or negative urgency (e.g., Shen, Lee, and Chen 2014; Wilbertz et al. 2014). Although neither negative urgency nor compulsivity were related to SSRT, a reduced P3 effect in individuals with longer SSRTs confirmed our hypothesis and matches existing literature (e.g., Huster et al. 2020; Kenemans et al. 2023; Lansbergen et al. 2007). Further, we found larger N2 amplitudes on failed compared to successful stop trials, even after controlling for ERP overlap and second‐level effects in the respective time window. However, the N2 effect is challenging to interpret, and more research is needed to understand its role in action cancellation and interindividual differences.

Although longer SSRT was associated with lower P1 amplitudes in successful and failed stop trials and later P3 latencies, negative urgency was related to a higher stop‐signal‐related P1 amplitude in successful stop trials and earlier P3 onset and peak. Thus, early stopping processes and the speed of initiating the action cancellation highlight interesting differences between negative urgency and SSRT. This emphasizes the importance of timing (Huster et al. 2020) and sensorimotor or attentional processes that interact with neural markers of inhibition (Hampshire and Sharp 2015). Given that larger P1 amplitudes on successful stop trials predicted earlier P3 onset and peak latency, the reduced stop‐signal‐related P1 effect (successful vs. failed stop) in negative urgency suggests a more efficient implementation of the action cancellation process. The successful stop‐signal‐related P1 amplitude showed significant incremental value over P3 onset, peak latency, and amplitude in explaining variance in negative urgency (see Supporting Information: Section B), further demonstrating the relevance of these early processes. This emphasizes that one strength of our study was examining the entire inhibition process instead of focusing on isolated markers.

The observed associations between negative urgency and neural effects may reflect a compensatory mechanism: Early processes and faster implementation of action cancellation compensated for potential reduced inhibitory activity, preserving task performance. These findings might suggest that several components interact synergistically in enhancing inhibitory control. This aligns with the process model of self‐control fatigue (Inzlicht and Schmeichel 2012), which posits that shifts in attention, motivation, or emotion can either contribute to enhanced self‐control or, conversely, result in self‐control failures. Higher expressions of self‐reported negative urgency would imply that these individuals are aware of their everyday life problems in inhibitory control. Greenhouse and Wessel (2013) reported larger N1 amplitudes when stopping (compared to go) was rewarded in the SST, suggesting that motivation facilitated inhibition through early preparation of the cancellation process. Similarly, in our study, participants with higher negative urgency might have been more motivated to prioritize stopping over going. Supporting this, individuals with higher negative urgency showed prefrontal hyperactivation during a go/no‐no task (Chester et al. 2016). However, excessive inhibitory control resulted in more alcohol use in the long‐term (Chester et al. 2016). Thus, the compensatory over‐recruitment of inhibitory control may deplete self‐control capacities (Baumeister, Vohs, and Tice 2007) or prove unsustainable over longer time. Finally, in accordance with the definition of negative urgency, impairments in inhibitory control may be limited to situations involving negative emotions.

Regarding compulsivity, most hypotheses had to be rejected. While the association between compulsivity with an earlier peak of the P3 did not withstand corrections for multiple testing, the direction of effects contradicted our hypothesis and findings in OCD (Kim et al. 2006). However, effects were in the same direction as negative urgency. This aligns with the conceptualization of negative urgency, which suggests that inhibitory control problems in the context of negative emotions give rise to impulsive and compulsive behaviors, thereby linking both traits (Zorrilla and Koob 2019). Thus, the irresistible urge to persistently perform actions that may lead to negative consequences is connected to both negative urgency and compulsive behavior. Although impulsivity and compulsivity were not correlated in our sample, compulsivity was correlated with negative urgency as well as other facets of impulsivity (lack of perseverance and lack of premeditation; see Table S1). As such, negative urgency may have accounted for the variance related to compulsivity in successful inhibition effects. Moreover, an increasing body of research supports a central role of negative urgency in addiction (e.g., Um et al. 2019) and as a transdiagnostic marker (e.g., Cyders, Coskunpinar, and VanderVeen 2016; Wolitzky‐Taylor et al. 2016). Future studies on OCD or repetitive behaviors should therefore examine whether failures in action cancellation are driven by negative urgency specifically rather than compulsivity or overall impulsivity.

### 4.3 General Limitations and Future Directions

The results need to be understood in the context of several limitations. We did not assess other potentially relevant markers, such as motor responses and beta bursts (Jana et al. 2020; Raud and Huster 2017), that could provide meaningful insights into the process of action cancellation. Some of our effects were relatively small, but considering biopsychosocial models, biological effects are expected to be modest because they only account for portions of the variance (Lehman, David, and Gruber 2017). It would have been beneficial to distinguish negative from positive urgency (Cyders et al. 2007), but an appropriate scale in German was not available at the time of data acquisition. Finally, it would be interesting to study the transition of functional impulsivity to problematic impulsive behavior in longitudinal studies.

### 4.4 Conclusion

This study revealed that (a) early sensorimotor or attentional activity (higher P1 amplitude for successful stop trials) appeared to facilitate action cancellation through a feedforward process, accelerating its neural implementation (earlier P3), and (b) negative urgency and SSRT exhibited distinct associations with the expressions of this mechanism. In the case of higher negative urgency, but not SSRT, the P1 amplitude that was specifically enhanced on successful stop trials and subsequently faster inhibitory recruitment (P3 onset and peak) may have compensated for lower inhibitory activity, as reflected in a diminished inhibition P3 effect (successful stop vs. go). These differences offer an explanation why these impulsivity facets show inconsistent associations. Taken together, our findings speak in favor of a complex and dynamically interacting model of action cancellation, with different sources for interindividual differences.

## Author Contributions

**Verena Wüllhorst:** conceptualization, formal analysis, investigation, methodology, visualization, writing – original draft. **Raoul Wüllhorst:** methodology, visualization, writing – review and editing. **Rebecca Overmeyer:** investigation, project administration, writing – review and editing. **Tanja Endrass:** conceptualization, funding acquisition, methodology, project administration, resources, software, supervision, writing – review and editing.

## Conflicts of Interest

The authors declare no conflicts of interest.

## Supporting information


Data S1.

## Data Availability

The data that support the findings of this study are available from the corresponding author upon reasonable request.
