# Supplementary material for: Comprehensive Analysis of Event‐Related Potentials of Response Inhibition: The Role of Negative Urgency and Compulsivity
Source: Psychophysiology. 2025 Feb 4;62(2):e70000. doi: 10.1111/psyp.70000 (PMC11794679; doi:10.1111/psyp.70000)
Supplement: Supplementary file 1 — Data S1. [file PSYP-62-e70000-s001.docx]

**Supplementary material**

**Contents**

[**A)** **Impulsivity as an overall score** 2](#_Toc176467768)

[**B)** **Exploratory analyses** 4](#_Toc176467769)

# **Impulsivity as an overall score**

**Table S1.** *Descriptive statistics of UPPS facets, an overall impulsivity score, and compulsivity as well as associations among these self-report scales and SSRT*

|  | **mean (SD)**  **[range]** | **urge** | **lprem** | **lpers** | **sens** | **comp** | **imp** | **SSRT** |
| --- | --- | --- | --- | --- | --- | --- | --- | --- |
| **urge** | 26.52 (5.91)  [14-44] | 1 | **.288** | **.424** | .015 | **.196** | **.529** | -.068 |
| **lprem** | 22.33 (4.33)  [13-38] | . | 1 | **.206** | **.325** | **-.236** | **.573** | -.103 |
| **lpers** | 19.50 (4.57)  [10-34] |  |  | 1 | -.016 | .053 | **.515** | -.032 |
| **sens** | 33.02 (6.94)  [14-46] |  |  |  | 1 | -.009 | **.207** | -.016 |
| **comp** | 13.08 (9.61)  [0-46] |  |  |  |  | 1 | .035 | .018 |
| **imp** | 60.77 (8.98)  [38-96] |  |  |  |  |  | 1 | -.071 |

*Note*. Urge = UPPS urgency, lprem = UPPS lack of premeditation, lpers = UPPS lack of perseverance, sens = UPPS sensation seeking, comp = compulsivity OCI sum score, imp = impulsivity BIS sum score, SSRT = stop signal reaction time. On the right, correlation coefficients. Significant correlations after Bonferroni-Holm correction are depicted in bold.

**Neural effects of overall impulsivity on successful inhibition (sStop vs. sGo)**

We conducted second-level regression analysis to investigate whether impulsivity, represented as a sum score of the BIS, exhibits similar effects on successful inhibition as negative urgency did. Results revealed that there was a negative-going effect between 664 - 778 ms at Cz, indicating that a higher overall impulsivity score was associated with a less pronounced successful inhibition-P3 effect, see Figure S1, but later than the effect of negative urgency was. There were no significant associations with the P1 amplitude, P3 onset or peak latencies, but a trend for an association with the P3 peak at Cz (*rho* = -.13, *p* = .059), in the same direction as we have seen in negative urgency.

**Figure S1.** *Second-level effects of overall impulsivity on successful inhibition effects*


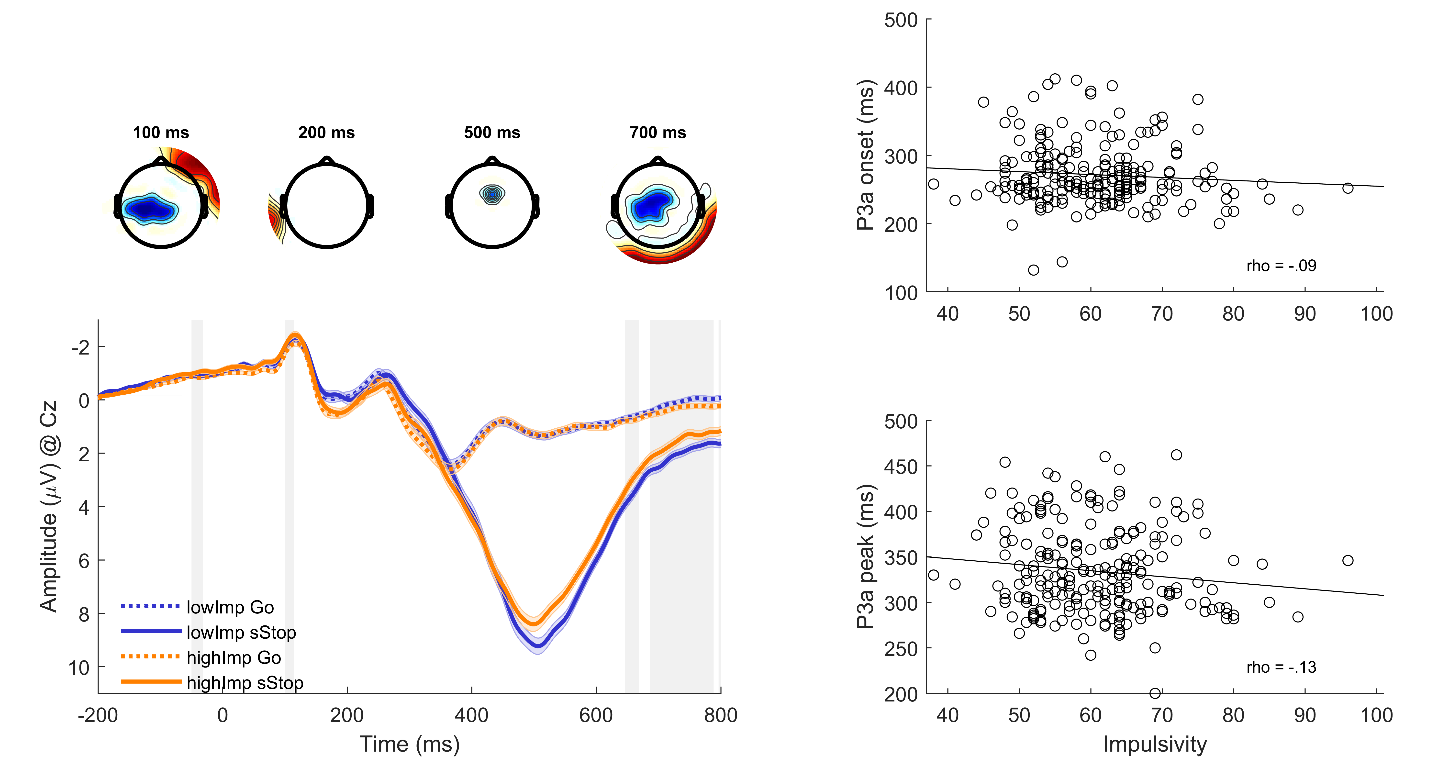


*Note.* Second-level effects of impulsivity on the successful inhibition effect at Cz. On the upper left, topographical maps represent significant associations between first-level effects and the regressor in question (t values, red: positive, blue: negative, masked at a corrected *p* = .05). Below, time courses of go-locked EEG activity (successful inhibition effect) are shown (for visualization purposes, waveforms are plotted for low vs. high impulsivity (Imp) after median split). sStop = successful stop trials. Gray shades reflect significance at the respective critical *p*. On the right, scatter plots show the non-significant associations of impulsivity with P3 onset and peak latencies at Cz. Critical *p* was .05.

# **Exploratory analyses**

**Exploration of expectancy effects**

We explored whether variations in stopping- P1 effects might be influenced by expectations, thus distinguishing this effect from preparation-related activity. We set up a first-level model with the formula:

$EEG= \beta_{0}+ \beta_{1}*stop trial lag+Error$

*Expectation control model.* In this model ‘stop trial lag’ (number of trials since the last stop-trial, parametric) served as the main predictor of interest in both failed and successful stop trials.

We observed effects of ‘stop trial lag’ on EEG activity, seen in positive-going EEG activity (CPz, peak at 294), form 206 - 410 ms after the stop trial, i.e. reflecting the P3, with more positive amplitudes in trials with a larger lag compared to a smaller lag to the last strop trial, *t*(232) = 1.11, *p* < .001, see Figure S2.

**Figure S2.** *Effect of stop trial lag on EEG activity*

**
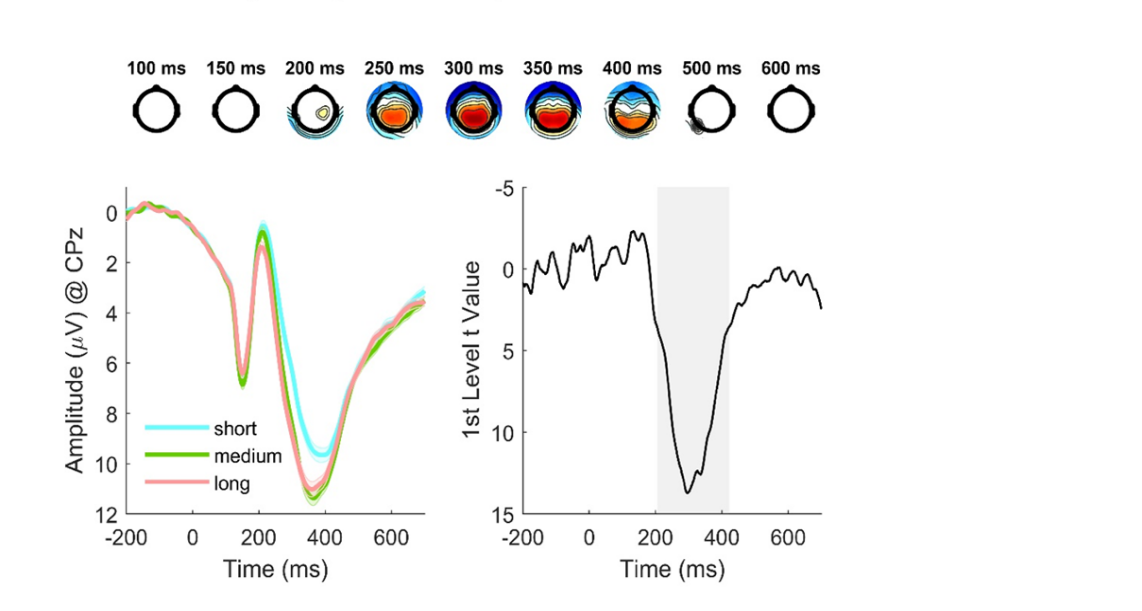
**

*Note.* Stop-locked task effects at CPz. Depiction of the parametric regressor ‘stop trial lag’ that incorporates the number of trials since the last stop trial. At the top, topographical maps represent significant associations between EEG activity and the regressor in question (*t* values, red: positive, blue: negative, masked with the fdr correction started at *p* = .001). Time course of stop-locked EEG activity is represented at the left side. For visualization, the plot shows EEG data after splitting the data in three bins resulting in a short, medium and long ‘stop trial lag’, e.g. one would indicate a short lag, whereas five trials were characteristic for a longer time lag. At the right side, trajectories of *t* values for the regressor are shown (gray shades reflect significance at *p* = fdr corrected).

**Exploration of associations of inhibition and stopping-related ERPs on negative urgency**

We examined whether the early stopping-P1 effect explained additional variance in negative urgency beyond the successful inhibition-related ERPs by comparing two multiple regression models. We selected relevant inhibition-related ERPs by using in each case the highest effect in previous analyses, resulting in the P3 onset at Pz, P3 peak and amplitude at Cz. As such, we were also able to investigate which of the predictors explained most variance in negative urgency.

As shown in Table S2, we observed that not only Model 1 containing the inhibition related ERPs significantly explained variance on urgency compared to a constant model, *F* (1,205) = 5.77, *p* < .001, but also Model 2, in which we added the stopping-P1 amplitude as an additional predictor, *F* (1,205) = 5.54, *p* < .001. Adding the mean amplitude of the P1 as a predictor significantly increased the explained variance on negative urgency by a change in *R*² = .021, *F* = 4.74, *p* < .05. Interestingly, the effect of the P3 amplitude was only significant, when also the predictor P1 amplitude was added in Model 2. We found negative regression coefficients for the P3-related predictors on negative urgency, in contrast to a small but positive effect for P1 amplitude.

**Table S2.** *Regression coefficients of inhibition and stopping related ERPs on negative urgency.*

| Variable | Model 1 | | | | | | | | | |  | | Model 2 | | | | | | | |  |  |
| --- | --- | --- | --- | --- | --- | --- | --- | --- | --- | --- | --- | --- | --- | --- | --- | --- | --- | --- | --- | --- | --- | --- |
|  | *B* | | β | | *SE* | | *t* | | *p* | | |  | *B* | β | | *SE* | | *t* | | *p* | |  |
| (Intercept) | 45.35 | |  | | 5.09 | | 8.91 | | .00 | | |  | 42.55 |  | | 5.21 | | 8.17 | | .00 | |  |
| P3 onset | -.03 | | -.21 | | .01 | | -2.63 | | .01 | | |  | -.03 | -.21 | | .01 | | -2.57 | | .01 | |  |
| P3 peak | -.03 | | -.21 | | .01 | | -2.56 | | .01 | | |  | -.02 | -.17 | | .01 | | -2.04 | | .04 | |  |
| P3 amplitude | -.14 | | -.13 | | .09 | | -1.54 | | .12 | | |  | -.19 | -.18 | | .09 | | -2.03 | | .04 | |  |
| P1 amplitude |  | |  | |  | |  | |  | | |  | .40 | .16 | | .01 | | 2.18 | | .03 | |  |
| *R*² | .077** | |  | |  | |  | |  | | |  | .098** | | |  | |  | |  | | |
| Δ *R*² |  |  | |  | |  | |  | |  | | | .021** | |  | |  | |  | |  |  |

*Note.* In Model 1, we examined the impact of the inhibition related ERPs (P3 onset, P3 peak, P3 amplitude) on negative urgency. In Model 2, we entered additionally the stopping-related P1 amplitude (at CP1) as a predictor. All predictors refer to successful stop trials. * *p* < .05, ** *p* < .001.

**Influence of trial difficulty (SSD) on EEG activity**

To test whether stop-signal-related P1 effects from the successful vs. failed inhibition model (2) can be explained in terms of SSD differences and/or overlap with go-related EEG activity, we compared EEG activity for short (easy trials) and long (difficult trials) SSDs after adjusting with unfold. We created a (unfold adjusted) short SSD subset, comprising trials with SSDs <= 150 ms, and a (unfold adjusted) long SSD subset, comprising trials with SSDs >= 200 ms (n = 3 participants did not fulfil stop accuracy requirements on long SSD trials), and compared P1 amplitudes. Results revealed larger P1 mean amplitudes at CP1 on long compared to short SSD trials for both stopping conditions: successful stop trials, *t*(461) = -3.70, *p* <.001 (*M*(short SSD) = 3.66, *M*(long SSD) = 4.64), and on failed stop trials, *t*(461) = -2.13, *p* <.05 (*M*(short SSD) = 5.78, *M*(long SSD) = 6.38), see Figure S3. Thus, a SSD effect was present in adjusted EEG data.

**Figure S3.** *EEG curves of successful and failed stop trials after correcting for go and motor overlap separately for short and long SSD trials*

**
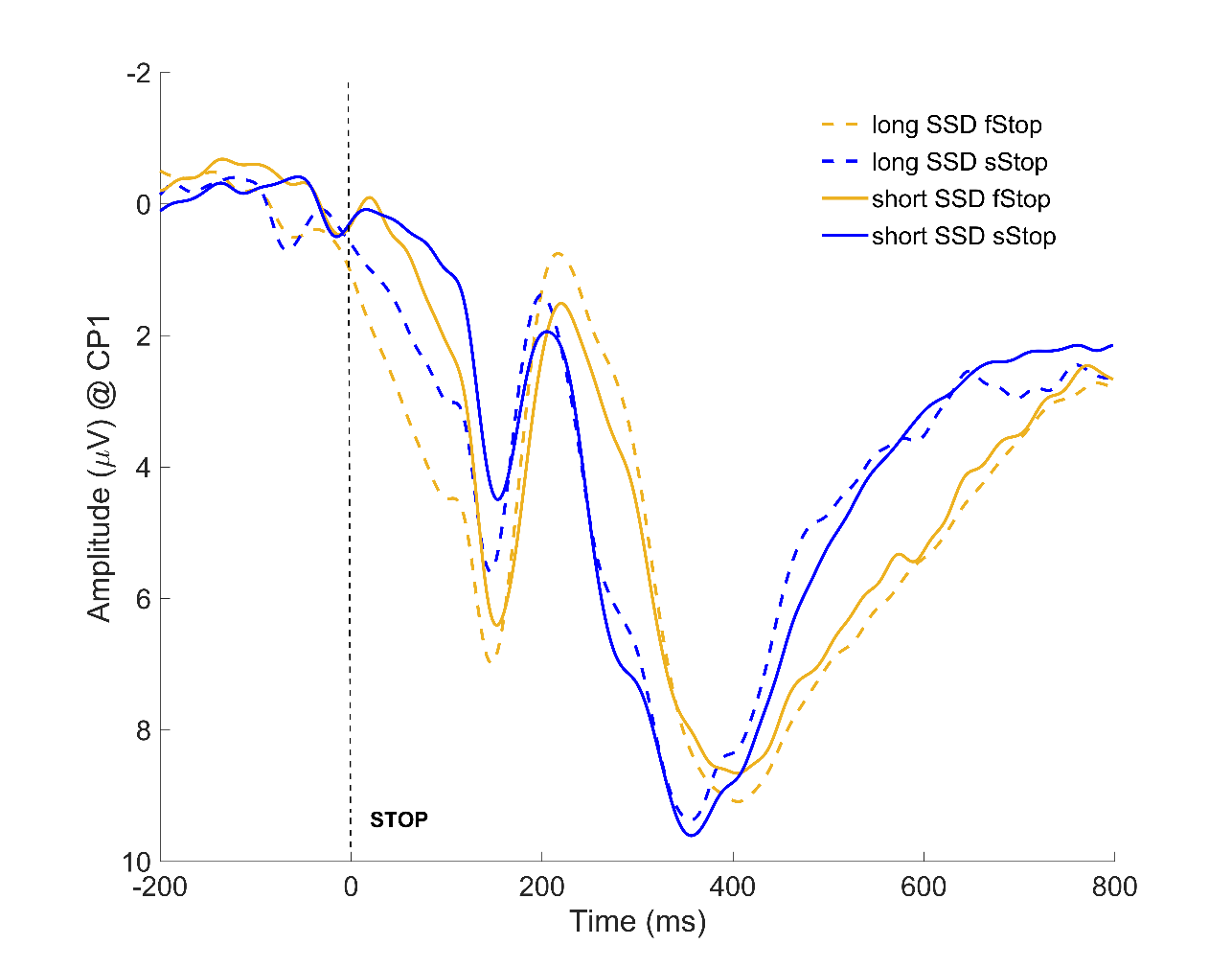
**

*Note.* fStop, failed stop trials, sStop, successful stop trials, SSD, individual stop signal delay at the end of the task.
